# Wellness in Nursing Education to Promote Resilience and Reduce Burnout: Protocol for a Holistic Multidimensional Wellness Intervention and Longitudinal Research Study Design in Nursing Education

**DOI:** 10.2196/49020

**Published:** 2023-09-08

**Authors:** Kelley Strout, Rebecca Schwartz-Mette, Jade McNamara, Kayla Parsons, Dyan Walsh, Jen Bonnet, Liam M O'Brien, Kathryn Robinson, Sean Sibley, Annie Smith, Maile Sapp, Lydia Sprague, Nima Sajedi Sabegh, Kaitlin Robinson, Amanda Henderson

**Affiliations:** 1 School of Nursing University of Maine Orono, ME United States; 2 Department of Psychology University of Maine Orono, ME United States; 3 School of Food and Agriculture University of Maine Orono, ME United States; 4 Office of Research Development University of Maine Orono, ME United States; 5 Department of Mathematics and Statistics Colby College Waterville, ME United States; 6 Electrical and Computer Engineering University of Maine Orono, ME United States

**Keywords:** nursing workforce, academic performance, burnout, resilience, wellness, nursing, education, nursing education, protocol, nursing students, students, holistic, implementation, workforce

## Abstract

**Background:**

The United States faces a nursing shortage driven by a burnout epidemic among nurses and nursing students. Nursing students are an integral population to fuel the nursing workforce at high risk of burnout and increased rates of perceived stress.

**Objective:**

The aim of this paper is to describe WellNurse, a holistic, interdisciplinary, multidimensional longitudinal research study that examines evidence-based interventions intended to reduce burnout and increase resilience among graduate and undergraduate nursing students.

**Methods:**

Graduate and undergraduate nursing students matriculated at a large public university in the northeastern United States are eligible to enroll in this ongoing, longitudinal cohort study beginning in March 2021. Participants complete a battery of health measurements twice each semester during the fourth week and the week before final examinations. The measures include the Perceived Stress Scale, the Satisfaction with Life Scale, the Oldenburg Burnout Inventory, the Brief Resilience Scale, and the Pittsburgh Sleep Quality Index. Participants are eligible to enroll in a variety of interventions, including mindfulness-based stress reduction, mindful eating, fitness training, and massage therapy. Those who enroll in specific, targeted interventions complete additional measures designed to target the aim of the intervention. All participants receive a free Fitbit device. Additional environmental changes are being implemented to further promote a culture that supports academic well-being, including recruiting a diverse student population through evidence-based holistic admissions, inclusive teaching design, targeted resilience and stress reduction workshops, and cultural shifts within classrooms and curricula. The study design protocol is registered at Open Science Framework (DOI 10.17605/OSF.IO/NCBPE).

**Results:**

The project was funded on January 1, 2022. Data collection started in March 2022. A total of 267 participants have been recruited. Results will be published after each semester starting in December 2023. WellNurse evaluation follows the Rapid Cycle Quality Improvement framework to continuously monitor ongoing project processes, activity outcomes, and progress toward reducing burnout and increasing resilience. Rapid Cycle Quality Improvement promotes the ability to alter WellNurse interventions, examine multiple interventions, and test their effectiveness among the nursing education population to identify the most effective interventions.

**Conclusions:**

Academic nursing organizations must address student burnout risk and increase resilience to produce a future workforce that provides high-quality patient care to a diverse population. Findings from WellNurse will support evidence-based implementations for public baccalaureate and master’s nursing programs in the United States.

**International Registered Report Identifier (IRRID):**

DERR1-10.2196/49020

## Introduction

### Background

The COVID-19 pandemic exacerbated already prominent feelings of burnout and stress among nursing students in the United States. Nursing students experienced high levels of perceived stress elicited by numerous interpersonal, intrapersonal, and community factors [1]. Nursing students’ stress contributes to less academic engagement [2], and over 55% of nursing students report increased stress and worsened health after entering nursing school [3]. During the COVID-19 pandemic, economic strain, academic workload, and isolation from peers accelerated nursing students’ stress and well-being [4]. Students cite limited clinical experiential learning and adjustment to web-based learning as prominent barriers influencing their intention to join the nursing workforce [4]. McKee-Lopez et al [5] found that adverse childhood experiences also contribute to mental health status among the nursing population, with nursing students experiencing a higher prevalence of adverse childhood events. Adverse childhood events have been associated with higher rates of burnout and depression among undergraduate nursing students [5]. Urban et al [6] found that nursing undergraduate students reported significantly higher stress levels than their nonnursing major peers. Moreover, COVID-19 can be seen as an adverse experience faced by all new incoming nursing students. A total of 60% of nurses younger than 35 reported an emotional, traumatic, or stressful event related to COVID-19 [7]. Nursing students are a critical component of the future nursing workforce. Currently, 55% of the nursing workforce experiences symptoms of burnout [8], contributing to an alarming acceleration of nurse turnover and shortages nationally. Stress does, in fact, predict nurses’ job satisfaction and turnover intention [9]. In total, 25% of nurses left the profession in 2022 compared with 20% in 2020, and the average hospital turned over 95% of its nursing staff in the past 5 years [10].

Personal resilience, or the capacity to adjust positively to adversity to limit negative impacts, has been correlated with reduced burnout [6]. As the United States faces a critical shortage of nurses confounded by high rates of burnout and stress, nursing schools must develop interventions and strategies to reduce burnout and promote resilience among nursing students. Stress reduction interventions could promote improved stress management and mitigate burnout among students [2,3,11], which can support nursing student retention, on-time graduation, and resilience when students transition to the nursing workforce.

The health care profession’s burnout crisis prompted the National Academy of Sciences to convene a committee and create a report that promotes the collective urgency to address burnout and resilience among health professionals, including nurses and students. Their vision includes a thriving health care workforce that provides high-quality care to patients in an environment that fosters their well-being [8]. The research study and project presented in this paper align with five of the seven priorities outlined by the academy: (1) create and sustain positive work and learning environments and culture; (2) invest in measurement, assessment strategies, and research; (3) support mental health and reduce stigma; (6) institutionalize well-being as a long-term value; and (7) recruit and retain a diverse and inclusive health workforce. There is a lack of substantive research that applies longitudinal analysis, large sample sizes, or randomization to support effective interventions that reduce burnout and promote resilience among nursing students [12], which this study aims to address.

WellNurse (Wellness in Nursing Education to Promote Resilience and Reduce Burnout) is a longitudinal research study implemented at a public nursing school in the northeastern United States to reduce burnout and increase resilience among nursing students. This multidimensional study examines the effect of various interventions on burnout, resilience, perceived stress, and academic performance. This paper aims to describe the comprehensive and holistic interventions and measures provided to nursing students at a public university in the northeast offering BSN and MSN education.

### WellNurse Project Goals

The project’s overarching purpose is to reduce burnout and increase resilience among nursing students at a public university in the northeast. Several objectives were implemented to achieve this aim, including (1) providing access to evidence-based mindfulness-based stress reduction (MBSR) to nursing students, faculty, and staff within a 3-year time frame; (2) providing evidence-based physical fitness, nutritional, and peer mentor training and support to all nursing students, faculty, and staff; (3) establishing a resilience and stress management curriculum to sustain program activities after the grant period with a credit-bearing course; and (4) creating an environment and culture that embraces resilience, wellness, diversity, equity, and inclusion. The study design protocol is registered at Open Science Framework [13].

## Methods

### Participant Recruitment

Matriculated graduate and undergraduate nursing students, faculty, and staff older than 18 years who complete a web-based consent form are eligible to participate in the study. The initial phase of the study occurred from March to May 2022, and recruitment is ongoing as new students and faculty will enter the program through December 2024.

The recruitment strategy involves the WellNurse research team visiting various in-person and remote nursing classes and hosting meetings with administrative staff and faculty. The team also posts recruitment messages on the school’s social media pages. Participants are offered a free Fitbit and enrollment in various interventions described below and can choose whether or not they consent to the evaluation aspect of the project. Some academic courses provide course credit, extra credit, or clinical hours to students who agree to participate. Faculty and staff are provided work time to participate in the interventions.

### Measures

#### Comprehensive Wellness Survey

After completing a web-based consent form, participants complete a comprehensive health behavior survey. This survey is distributed during week 4 and again during week 14 (the week before finals week) in the fall and spring semesters. Week 4 was selected based on the typical nursing student’s academic loads and stress. Examinations and competency testing begin each semester’s third to fourth week, accelerating stress during those periods. Participants complete the comprehensive health behavior survey during designated class or work time. Additional measures are collected before specific engagement in targeted study interventions. Participants’ answers are tracked through the project using unique ID codes. As listed below, several variables are assessed to capture the health of nursing students. The comprehensive measurement strategy aligns with Priority Area 2 from the National Academy of Sciences [8], which recommends the longitudinal use of valid and reliable measurements of burnout and factors that mitigate burnout to drive effective interventions and data-driven decision-making at the organizational level.

##### Demographics

Participants are asked several sociodemographic questions, including gender identity, sexuality, and ethnicity. Participants also provide their grade point average. Student progression status is also tracked to determine how various interventions influence retention and on-time graduation.

##### Perceived Stress Scale

The Perceived Stress Scale (PSS) is used to measure perceived stress. The scale includes 10 items that address experiences of distress related to “how unpredictable, uncontrollable, and overloaded respondents find their lives” [14]. Example items include “In the last month, how often have you felt that you were unable to control the important things in your life?” and “In the last month, how often have you felt difficulties were piling up so high that you could not overcome them?” The PSS demonstrates reliability and validity among healthy college populations [14].

##### Satisfaction With Life Scale

The Satisfaction with Life Scale is used to measure satisfaction with life. The 5-item Satisfaction with Life Scale [15] uses Likert scale responses ranging from “strongly agree” to “strongly disagree.” An example item is “The conditions of my life are excellent.” The measure demonstrates strong validity and reliability in past research [16,17].

##### Oldenburg Burnout Inventory

The Oldenburg Burnout Inventory is a 16-item measurement tool that measures the concepts of disengagement and exhaustion to assess burnout. Participants rank statements such as “When I work, I usually feel energized,” with Likert responses ranging from “strongly agree” to “strongly disagree.” The Oldenburg Burnout Inventory is a valid and reliable measure of burnout related to work and academics [18].

##### Brief Resilience Scale

The Brief Resilience Scale is a 6-item scale that measures resilience and one’s ability to bounce back and recover from stress. The Brief Resilience Scale is a valid and reliable measure of resilience [19].

##### BMI

BMI screens for weight categories that may be associated with health problems and reflects a person’s weight in kilograms divided by the square of height in meters [20]. Participants were asked to self-report their height and weight, and a BMI for each participant was derived accordingly.

##### Pittsburgh Sleep Quality Index

Sleep quality is measured using the 2-item Pittsburgh Sleep Quality Index [21], which assesses participants’ estimated average hours of sleep per night and overall perception of sleep quality within the past 7 days. This survey has demonstrated acceptable validity and reliability in past research [21].

#### Nutrition Assessments

WellNurse participants have an opportunity to engage in a mindful eating cooking workshop conducted by a registered dietitian nutritionist. Before attendance, participants are required to complete a web-based registration form and relevant assessments. The assessments involve multiple health and nutrition behavior surveys, described below.

##### Mindful Eating Questionnaire

The Mindful Eating Questionnaire was developed to measure mindful eating, which can be described as nonjudgmental awareness of physical and emotional sensations associated with eating [22]. This 28-item scale comprises several subdomains, including disinhibition, awareness, external cues, emotional response, and distraction. Scores range from 0 to 4, with higher scores indicating greater mindful eating skills. This scale has been previously validated in adult populations [22].

##### Food Preparation Experience and Confidence Survey

This survey measures cooking self-efficacy and frequency of use of 13 different cooking styles (eg, boiling, baking, and microwaving). Participants are asked to rate their perceived confidence of each cooking method, ranging from confident, to uncertain, to not confident. This survey has been validated among young adults [23].

##### Body Appreciation Scale

Body appreciation reflects positive body image and has been previously associated with higher levels of self-esteem and lower levels of coping by avoidance. The Body Appreciation Scale is composed of 13 items, to which participants are asked in a Likert format the frequency to which they exhibit each statement [24]. Examples of statements on the questionnaire include “I respect my body” and “I do not focus a lot of energy being concerned with my body shape or weight.” Scores range from 1 to 5, with higher scores indicating higher levels of body appreciation. This scale has been validated among young adults [24].

##### Weight Satisfaction

Weight satisfaction is measured using 3 items. The first asks, “Which of the following are you trying to do about your weight?” This gives respondents the ability to state that they are trying to lose weight, gain weight, stay the same weight, or that they are not doing anything about their weight. The second item measures how they feel about their current weight, with responses including “happy,” “upset,” or “don’t care.” The third item inquires about their desired weight in pounds. Weight satisfaction is then determined using the formula: [(actual weight – desired weight)/actual weight × 100].

##### Short Healthy Eating Index

Diet quality is measured using the Short Healthy Eating Index, validated in college populations [25]. Higher scores indicate greater adherence to the Dietary Guidelines for Americans, 2020-2025 [26].

#### Physical Activity Assessments

WellNurse participants have an opportunity to engage in physical fitness training to prepare for a 5K walk or run. Before and after the training, participants complete a comprehensive measure of physical activity.

##### International Physical Activity Questionnaire

The International Physical Activity Questionnaire measures participants’ frequency of physical activity [27]. This 4-item instrument provides an overall estimate of physical activity levels over a week. The measure is valid and reliable in assessing college students’ physical activity [28].

##### Fitabase Data Collection

Participants also have an opportunity to receive a free Fitbit Versa 2 (Fitbit). Because various health indices can be derived from ongoing data collection metrics within the Fitbit, participants complete an in-person consent form separate from the WellNurse consent form before receiving their device. The WellNurse research team connects each Fitbit to the web-based database, *Fitabase*. Fitabase tracks a multitude of movement and health-related behaviors, including steps taken per day, distance traveled, calories burned per day, heart rate variability (sedentary-very active minutes, logged physical activity, floors, basal metabolic rate, and resting heart rate), and sleep behavior (minutes in light sleep, deep sleep, and rapid eye movement sleep).

#### Mindfulness-Based Stress Reduction

Participants who sign up to participate in MBSR complete additional pre- and postintervention measures.

##### Five Facet Mindfulness Questionnaire

Mindfulness is measured using the Five Facet Mindfulness Questionnaire. This survey is composed of 39 questions and five subscales, including (1) observing, (2) describing, (3) acting with awareness, (4) nonjudging, and (5) nonreactivity. This survey has demonstrated good validity and reliability in past research [29].

##### Positive and Negative Affect Scale

Participants are asked to complete the 20-item Positive and Negative Affect Scale, which measures underlying experiences of positive or negative feelings of mood or emotion. This survey has demonstrated excellent validity and reliability in past research [30].

#### Massage Therapy

##### Short Form PSS

The Short Form PSS is a 4-item perceived stress survey that demonstrates validity and reliability [31]. Participants who receive a massage are invited to complete the Short Form PSS before the massage session and again 1 week later. Additionally, they have an option to complete a qualitative question on the follow-up survey, “Describe how massage therapy has affected your stress, anxiety, or happiness. Use specific examples if possible.”

#### Attendance Records

When participants attend any additional workshop or activity, such as an exercise class, massage, or use of the WellNurse room, they sign in to track attendance.

### Interventions Under Investigation

Holistic and comprehensive interventions are under investigation that align with 4 of the National Academy of Health’s priorities areas: Priority 1: create and sustain positive work and learning environments and culture; Priority 3: support mental health and reduce stigma; Priority 6: institutionalize well-being as a long-term value; and Priority 7: recruit retain a diverse and inclusive health workforce [8].

### Environmental and System Interventions

#### Diversity, Equity, and Inclusion

According to the United States Census Bureau [32], individuals from ethnic and racial minority groups accounted for 40% of the population in 2022. Nursing programs have a responsibility to recruit and retain a more diverse student body and more diverse faculty and staff to build a vibrant nursing workforce that reflects the diversity of the population it serves [33]. Therefore, several institutional changes were implemented and are ongoing to promote a culture that embraces diversity, equity, and inclusion (DEI).

A DEI committee was established at the school in 2021. An extensive holistic admission review training was provided to the faculty and staff who interface with the admission process. A holistic admission review process was implemented that includes objective and relational admission measures. Training and workshops about diverse populations were implemented as part of routine team meetings and class presentations. Curricular experts offered courses and programming to promote faculty transforming their courses to align with Inclusive Teaching Design. Inclusive teaching is being responsive to the diversity of the class and designing learning environments that include all students while allowing students to be engaged in an equitable learning environment and feel a sense of belonging [34]. The design begins with a clear syllabus outlining tools needed for success in a course, and transparent assignment frameworks highlighting what the learner needs to do, why they need to do it, how they should do it, and how their work will be evaluated. Faculty also include an intersectionality statement in the syllabus to state their commitment to the collective diversity learners bring to the learning environment.

Classes also include a *Getting-to-Know-You Survey* before the first class meeting that identifies learner preferences and perceived barriers to learning for early intervention. The survey is used for targeted student outreach, with an initial email offering resources specific to their concerns and a frequently asked question response to all students for commonly asked questions. Building community is a focus of the first class meeting. Students introduce themselves and share pictures from their life on the learning management software discussion board. Faculty share their social identities with the class and discuss the contexts that impact learning and teaching. Vulnerability and reflexivity are essential to breaking down real and exaggerated power differentials in the course. Ground rules are discussed and published on the learning management system during the first class. A reflection prompt completed in the first meeting allows learners to consider how they manifest their nurse role. Establishing purpose with a sense of belonging and self-confidence is threaded throughout the class meetings. Holistic admissions practices and inclusive teaching strategy training from experts are incentivized among faculty and staff and ongoing.

The faculty who teach Psychiatric Mental Health Nursing implemented wellness and resilience strategies in their classrooms. Faculty provided students with several mindfulness and wellness classroom options, such as guided meditations; mindful movement and stretching; and information about campus resources before class, during times of transition, or after emotionally taxing lecture content such as suicide. Faculty also implemented short meditations before examinations and provided extra credit to engage in any of the WellNurse programming.

#### WellNurse Room

A conference room in the School of Nursing was converted to a WellNurse room. The WellNurse room signals an institutional and systemic investment in supporting the resilience of incoming students, faculty, and staff. The renovation also promotes recruitment into the MBSR program by raising the profile of these activities with dedicated space for practice.

Participants are invited to attend free workshops that aim to promote healthy behavior change through evidence-based practices. Each workshop is designed and led by an interdisciplinary member of the research team. Advertisements for upcoming workshops are posted to the School of Nursing’s social media pages and through email blasts. Attendance is optional. Workshops address various topics of concern, including the role of diet quality, sleep, and navigating test anxiety. Health behavior workshops are held monthly, both remote and in-person. Recordings are available to participants upon request.

### Classroom Enhancements

Faculty integrate selected WellNurse interventions into their classes by offering extra credit or providing assignments attached to interventions. Additionally, faculty use class time to implement short periods of mindfulness either through guided meditations, outdoor walks, or open discussion about how students might embrace mindfulness and self-care during a rigorous schedule.

### Wellness and Resilience Social Media Presence

A graduate student was hired to promote WellNurse activities and interact with students on social media. These posts highlight brief research findings about resilience, stress, and well-being among nurses and students. They also guide students through time management, test-taking strategies, community-building activities, and more.

### Mindfulness-Based Stress Reduction

MBSR is an 8-week experiential program including 2.5-hour weekly class sessions and a 1-day silent retreat. MBSR engages participants in simple evidence-based mindfulness practices, including meditation, mindful walking and movement, cognitive reframing, and communication strategies. Pioneered in 1979 by Dr Jon Kabat-Zinn at the University of Massachusetts Medical Center, MBSR includes didactic and experiential training that teaches participants to cultivate self-awareness by redirecting attention away from past or future concerns and focusing instead on the present moment. With regular practice, participants learn to change habitual stress reactivity patterns and instead more flexibly respond to stress with adaptive coping. As a result, participants learn to avoid internalizing stress in reactive patterns of negative emotion [35] and, thus, reduce distress and prevent negative health and mental health outcomes.

WellNurse partnered with the University of California San Diego School of Medicine Center on Mindfulness and Brown University Mindfulness Center to provide remote, 8-week MBSR courses via Zoom and intensive week-long, in-person MBSR courses. MBSR was offered as an elective course to count toward the students’ elective requirements for Nursing. Additionally, faculty and staff were provided work time to participate in the program. Selected WellNurse project personnel including faculty, staff, and graduate students completed a multiyear teacher training certification process through the University of California San Diego and Brown University Mindfulness Center to develop a group of certified MBSR teachers to support long-term project sustainability.

### MasterChef: Nursing Edition

This monthly workshop is innovative in combining gamified learning, cooking self-efficacy, mindful eating, and nutrition education for prelicensed nursing students. The course outline consists of (1) mindful eating concepts, (2) nutrition education, (3) participants designing their own healthful and accessible recipe, (4) participants mindfully eating and voting on their favorite meal, and (5) discussion and application to clinical practice. Participants are also encouraged to do 2 “homework” assignments per week, each emphasizing healthy eating or mindfulness behaviors. Participants also receive ingredients for recreating their healthful recipe at home and a “dorm-friendly” recipe book developed by a registered dietitian nutritionist. All students, staff, and faculty are encouraged to join, but students in designated courses receive credit for attendance.

### Fitness Interventions

Participants are eligible to receive a free Fitbit Versa 2. Fitness trainers provide weekly 5K training in the fall and spring semesters to prepare participants for a 5K walk or run. Additionally, the campus recreation center offers nursing-specific fitness courses such as yoga and full-body workouts to promote a fitness community. Faculty are integrating fitness step challenges using the Fitbit devices as part of class assignments.

### Ethics Approval

The University of Maine institutional review board reviewed and approved the WellNurse research project procedures (02-05-2-2022).

## Results

### Overview

The project was funded on January 1, 2022. Data collection started in March 2022. A total of 267 participants have been recruited. Results will be published after each semester starting in December 2023.

The primary study variables will be analyzed 4 times per year to track population outcomes and changes. Regression analysis will be conducted to examine the relationship of students’ on-time progression, stress, resilience, and burnout with physical and emotional health variables and on-time progression. Subanalysis will be conducted for unique groups such as first and fourth year students, faculty and staff, and graduate students, as well as students from historically underrepresented populations such as first-generation college students and racial and ethnic minorities. Additionally, program retention, on-time graduation, and faculty and staff retention will be tracked over time. The cost of implementing MBSR, massage therapy, MasterChef, and fitness events will be tracked to determine cost per student. Additionally, feasibility will be measured using qualitative focus group interviews with students who complete specific interventions along with attrition rates for completing specific interventions such as MBSR and MasterChef.

### MBSR Analysis

Quantitative evaluation of MBSR outcomes will be conducted by comparing MBSR participants with matched control participants at selected intervals with regard to key study variables (eg, stress, burnout, and mindfulness skills). Participants enrolled in MBSR are assessed immediately before their first MBSR session and immediately after completing the final MBSR session. Matched controls are identified for each MBSR participant by randomly selecting nonenrolled individuals from the WellNurse participant pool on the basis of identical gender identity, birth year, and role (faculty, staff, and student). Controls are assessed at the same intervals as MBSR participants.

Statistical analysis will test whether MBSR participants differ significantly from controls with regard to postintervention levels of each outcome variable, controlling for preintervention levels. ANOVA models will be used to conduct initial comparisons. With time, larger samples, and thus more statistical power and more complex models (eg, multilevel modeling), can be tested to evaluate the potential impact of instructor, delivery modality (in-person vs remote), duration (1 week vs 8 weeks), and dose (number of sessions attended), as well as individual participant characteristics (eg, gender identity and role). Ongoing assessments for all WellNurse participants will allow for longer term follow-up of MBSR and control participants with regard to distal outcomes of interest (eg, academic retention). We expect significant differences between MBSR and control participants; immediate postintervention effects may be strongest, with somewhat weaker longitudinal effects observed over time. Analyses will also be able to test whether those participants who engage in ongoing mindfulness practice after formal MBSR training may experience improved outcomes as compared with those individuals who discontinue regular practice.

### Physical Activity Analysis

Data logs generated from Fitbit devices will be collected from the Fitabase platform [36], a partner to Fitbit, and raw data will be preprocessed, including merging the data of all participants in the study and purging the data set from empty cells or invalid data. The privacy-respected data set, besides participants' ID and event date, involves information on 3 major categories—sleep, activity, and wearing time—with each category covering more information in their respective parameters for a duration of fall and spring academic semesters. The sleep category provides information ranging from sleep minutes in rapid eye movement, deep, light, and total asleep minutes to sleep efficiency. The activity category is reporting time and distances traveled in very active, moderately active, lightly active, and sedentary modes, alongside calories, basal metabolic rate, and approximated heart rate levels. Analysis of the data set is done using the Python language [37] and the Scikit-learn [38], Pandas [39], Numpy [40], Matplotlib [41], and Seaborn [42] packages by applying the multiple linear regression method and also generating a heatmap of the parameters.

### MasterChef Analysis

MasterChef will be evaluated initially for feasibility through a qualitative focus group design along with an expert content analysis. After modifications to the program based on feasibility analysis, the intervention will be evaluated using a matched-based cohort design with the non-MasterChef control group selected based on gender, ethnicity, and year in college matched-based design, with a focus on gender, ethnicity, and year in college. Descriptive and frequency statistics were used to analyze the sample’s baseline and demographic data. A 2-way repeated-measures analysis will be used to assess significant changes in baseline and postintervention outcomes within the experimental group and as a comparison with the control. Participants will complete the aforementioned nutrition measures before the week-long Fall 2023 program delivery, directly after the program, and again at the end of the semester. A control will be recruited through a matched-based design. A 2-way repeated-measures ANOVA will be used to identify significant changes between baseline data, postintervention data, and follow-up data between the intervention and control group. The program will also be implemented in the format of monthly workshops. A multivariate ANOVA will be used to compare health outcomes between curriculum formats. Multiple linear regressions will be used to identify predictors of diet quality using the combined fall and spring samples. Significance levels will be set at *P*<.05.

## Discussion

### Principal Findings

Stress and burnout among nurses and students is not a new phenomenon. However, the COVID-19 pandemic has exacerbated the challenge and demonstrated the urgency in mitigating stress to improve well-being and resilience and reduce burnout. As the United States faces a looming nursing shortage and burnout crisis, more research that includes large sample sizes and longitudinal studies is needed to determine and implement resilience-building and stress reduction interventions within nursing programs [12]. Interventions that demonstrate effectiveness at improving student retention, reducing the risk of burnout, and improving resilience and well-being will be infused into a course, which will be embedded as part of the standard nursing curriculum.

### Comparisons With Prior Work

#### WellNurse Room

Effective strategies to promote resilience and reduce burnout require system and environmental interventions [43]. The room contains floral murals, live plants, aromatherapy, meditation cushions, light therapy, coloring books, and tactile engagement. The WellNurse room is stocked with meditation cushions and a massage therapy table. Weekly mindfulness sessions are offered to support MBSR practices. A licensed massage therapist provides complimentary massage therapy in 15- to 30-minute sessions. Aromatherapy and massage therapy have shown stress-reducing effects [44,45]. This study aims to understand how environmental changes may influence nursing students’ resilience, stress, and well-being.

#### Mindfulness-Based Stress Reduction

Extensive literature supports MBSR as an effective intervention in reducing burnout among nurses [46-52].

MBSR, meditation, and mindful movement are further effective in treating posttraumatic stress disorder and depression symptoms [35], preventing substance use relapse, and curbing binge-eating behavior [53-55]. MBSR has demonstrated better outcomes for binge-eating than cognitive-behavioral therapy [56] and is as effective as anxiolytic medication in managing anxiety disorders [57].

College students with mindfulness training have been able to better resist the negative impact of stress during challenges [58], and mindful movement and meditation are both recommended as nonpharmacologic interventions to reduce stress and anxiety among college students [43]. Among medical students, these practices were found to improve happiness, self-confidence, endurance, and patience [59]. Systematic reviews have demonstrated that MBSR decreases stress and burnout and increases resilience, coping, and job satisfaction among nurses [60,61]. Given MBSR’s significant benefits with regard to physical and mental health and its demonstrated impact in nursing and college student populations, this evidence-based intervention was an ideal fit for the WellNurse nursing students. This study aims to examine the effect of MBSR on graduate and undergraduate nursing students’, faculty’s, and staff’s resilience, perceived stress, mindfulness, and burnout.

#### Nutrition

Nursing students report poor diet quality and dietary behaviors elicited by high levels of perceived stress, peer influence, and academic workload [62]. The impact of mindful eating may mediate this relationship [63]. Mindful eating describes applying mindfulness practices to internal hunger and satiety cues and incorporating a nonjudgmental, self-compassion–led viewpoint in navigating dietary choices [64]. Mindful eating interventions effectively improve several health behaviors, including reducing extreme dieting behaviors, improving portion control, and weight reduction within some populations [65,66]. This study aims to examine the effect of the newly developed program, MasterChef Nursing Edition, on nursing students’ diet quality, resilience, burnout, and well-being.

#### Fitness

Physical activity reduces stress and anxiety [67-70], and stress and anxiety are associated with increased burnout and decreased resilience among nurses [52]. A review of 37 studies on Fitbit-based interventions demonstrated the effectiveness of this wearable technology for physical fitness support; wearing a Fitbit significantly increased daily step counts and moderate to vigorous physical activity and significantly decreased weight [69]. Although students have access to healthy food and physical fitness environments, nursing programs do not systematically integrate support systems that promote physical well-being [12,71-74]. The aim of this study is to examine the effect of fitness interventions on nursing students’, faculty’s, and staff’s burnout, resilience, fitness, and well-being.

### Conclusions

WellNurse aims to examine multiple resilience-building and stress-reducing strategies while creating a culture that promotes student, faculty, and staff well-being in concert with academic and clinical preparedness and job satisfaction. Dissemination of findings will be released as they are available with an anticipated release date of initial findings in 2024.

## Abbreviations

DEI: diversity, equity, and inclusion
MBSR: Mindfulness-Based Stress Reduction
PSS: Perceived Stress Scale

## Data Availability

Data sharing is not applicable to this article as no data sets were generated or analyzed during this study.
